# Single-Stage Underwater Target Detection Based on Feature Anchor Frame Double Optimization Network

**DOI:** 10.3390/s22207875

**Published:** 2022-10-17

**Authors:** Huilin Ge, Yuewei Dai, Zhiyu Zhu, Xu Zang

**Affiliations:** School of Maine, Jiangsu University of Science and Technology, Zhenjiang 212003, China

**Keywords:** underwater object detection multi-scale, dynamic convolution, UWNet, compound connection network

## Abstract

Objective: The shallow underwater environment is complex, with problems of color shift, uneven illumination, blurring, and distortion in the imaging process. These scenes are very unfavorable for the reasoning of the detection network. Additionally, typical object identification algorithms struggle to maintain high resilience in underwater environments due to picture domain offset, making underwater object detection problematic. Methods: This paper proposes a single-stage detection method with the double enhancement of anchor boxes and features. The feature context relevance is improved by proposing a composite-connected backbone network. The receptive field enhancement module is introduced to enhance the multi-scale detection capability. Finally, a prediction refinement strategy is proposed, which refines the anchor frame and features through two regressions, solves the problem of feature anchor frame misalignment, and improves the detection performance of the single-stage underwater algorithm. Results: We achieved an effect of 80.2 mAP on the Labeled Fish in the Wild dataset, which saves some computational resources and time while still improving accuracy. On the original basis, UWNet can achieve 2.1 AP accuracy improvement due to the powerful feature extraction function and the critical role of multi-scale functional modules. At an input resolution of 300 × 300, UWNet can provide an accuracy of 32.4 AP. When choosing the number of prediction layers, the accuracy of the four and six prediction layer structures is compared. The experiments show that on the Labeled Fish in the Wild dataset, the six prediction layers are better than the four. Conclusion: The single-stage underwater detection model UWNet proposed in this research has a double anchor frame and feature optimization. By adding three functional modules, the underwater detection of the single-stage detector is enhanced to address the issue that it is simple to miss detection while detecting small underwater targets.

## 1. Introduction

Due to the complex underwater environment, the turbidity of the water body, the absorption of light by the water body, and the high cost of underwater video acquisition, machine vision still has much room for development in the field of aquatic biological monitoring. Underwater robots can realize the function of allowing robots to complete specific underwater tasks instead of manual diving. Underwater robots are widely used in safety search and rescue, pipeline inspection, oil exploration, and fishing.

**Figure 1:**
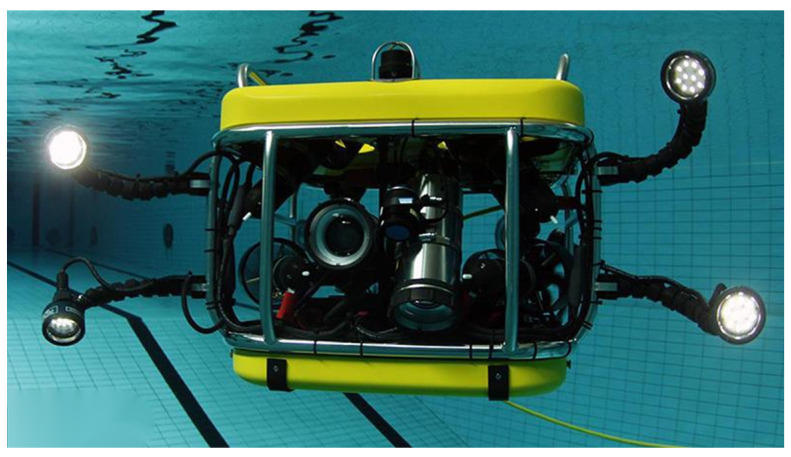
Current mainstream underwater robots.

The movement and operation of underwater robots are usually remotely controlled by operators on water ships and interact through vision and control systems [1]. Equipped with sonar, laser systems, cameras, and other equipment, real-time video and sonar images are provided by underwater robots for water operators. To grasp the target in the dark underwater environment, the underwater robot will also be equipped with a mechanical arm and a searchlight. However, it is not enough for underwater robots to achieve autonomous target grasping with the above equipment and sensors alone, and a set of underwater target detection algorithms are demanded. The current mainstream underwater robots are shown in Figure 1.

To accurately identify the target, the key is to determine the category and location of the underwater target. The most direct method is to collect images through underwater cameras and implement detection through the deployed underwater target detection algorithm [2]. However, the shallow aquatic environment is complex. Problems such as color shift, uneven illumination, blurring, and distortion would occur in the imaging process. These scenes are very unfavorable for the detection network. In addition, due to the existence of image domain offset, it is difficult for general object detection algorithms to maintain high robustness in underwater environments [3].

Today, underwater object detection still faces many challenges. For example, there are many small targets in the underwater scene due to suspended objects and uneven illumination in the underwater environment. Secondly, deploying underwater target detection algorithms on the mobile terminal requires high precision and lightweight. It is difficult to quantify the detection model, and it is difficult to reduce the weight of the detection model while maintaining high accuracy. In addition, the current stage of shallow sea underwater detection datasets has few types and minor scales, and it is urgent to expand the scale of existing underwater datasets.

In summary, the current underwater target detection is limited by the complex underwater environment, and the general detection algorithm has little effect, making underwater target detection still challenging. To promote the development of underwater robot technology and realize the “transparent ocean” as soon as possible, the research on underwater target detection is of great significance and value.

### 1.1. Related Work

**Figure 2:**
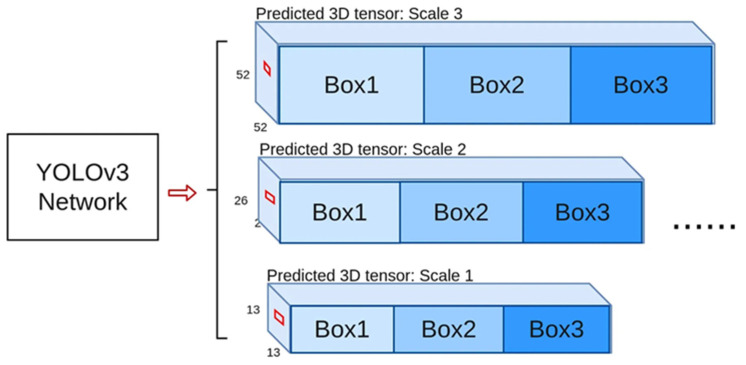
Yolov3 network structure diagram.

In terms of deep learning algorithms, the AlexNet model of Deep Convolutional Neural Network (DCNN) proposed by Krizhevsky [4] et al. in 2012 achieved record image classification accuracy at the IMAGENET Visual Recognition Challenge (ILSRVC) to obtain a classification model. Since then, deep learning has been widely used in recognition and detection with good results. In 2013, Ross Girshick [5] and others first applied the CNN method to the target detection task. They used the traditional image algorithm Selective Search to generate candidate regions and succeeded wildly. This region has a far-reaching influence on the target detection field—Convolutional Neural Network (R-CNN) model. In 2015, Ross Girshick improved this method and proposed the Fast R-CNN model. By sharing the calculation of the convolution layer for objects in different regions, the amount of analysis is significantly reduced, the processing speed is improved, and a regression method for adjusting the position of the target object is introduced, which further enhances the accuracy of position prediction. In 2015, Shaoqing Ren [6] et al. proposed the Faster R-CNN model and the RPN method to generate candidate regions of objects. This method no longer needs to create many candidate regions and further improves the processing speed. In the same year, J. Redmon [7] proposed a new object detection method, YOLO, which no longer uses the separation module of R-CNN. YOLO does not require the process of two detections and uses image detection as a regression problem to describe the space. Separate bounding boxes and associated class probabilities surpassed the then-hot R-CNN in speed and performance. After that, the author continued to improve, and Yolov2 [8] appeared in 2016. As of 2018, the author launched Yolov3 [9], which had better performance and was applied in more fields, especially in the military. The author of the YOLO series was afraid that the YOLO model would be improperly used. The YOLO series models are no longer updated. In 2017, Kaiming He [10] et al. proposed the Mask R-CNN model, which can simultaneously achieve the tasks of target detection and object instance segmentation by adding a relatively small amount of computation to the FasterR-CNN model. The network structure of Yolov3 is shown in Figure 2.

In terms of target detection applications based on deep learning, in 2019, Li Qingzhong [11] and others proposed a real-time detection algorithm for underwater fish targets based on improved YOLO and transferred learning. Furthermore, an underwater-YOLO network structure was constructed to rebuild the complexity of the deep learning network structure. The number of convolution layers and convolution kernels enables the model to realize real-time detection of underwater fish targets applied in the embedded system of underwater robots, and the fish detection accuracy rate reaches more than 93%. This method profoundly learns large scale. The network model can be used in embedded systems, which is in line with the development direction of the current technology transition from PC to mobile. By adding the train professionals generation network to the YOLOv3 detection network as its enhancement network, Liu Ping [12] and others proposed a marine biometric recognition algorithm in 2020. Compared with the traditional method of separating image processing and detection model, this method is more targeted for underwater detection and recognition. The application of the network model is more systematic and concise.

### 1.2. Our Contributions

This paper proposes a single-stage detector-based composite connection structure to aggregate the advantages of different backbone networks to enhance feature discrimination. To strengthen the ability of multi-scale detection, an improved ASPP+ module based on ASPP is introduced for multi-backbone intermediate connections. A receptive field enhancement module is further proposed to expand the feature sensory field area through deconvolution. Finally, since the positive and negative samples are imbalanced in single-stage detectors and the scarcity of underwater datasets exacerbates the imbalance, inequality draws on the design ideas of two-stage detectors. It proposes a refinement module, which simply implements a single-stage detector. In the pre-screening process of positive and negative samples of the stage detector, the anchor frame and features are corrected in the final stage to align the features and anchor frames, thereby significantly improving the accuracy of the single-stage detector based on introducing a small overhead.

The rest of the paper is organized as follows: Section 2 describes our methods and materials in detail. Section 3 presents the experimental results. Section 4 discusses them. Section 5 concludes.

## 2. Methods and Materials

### 2.1. Underwater Object Detection Dataset

**Figure 3:**
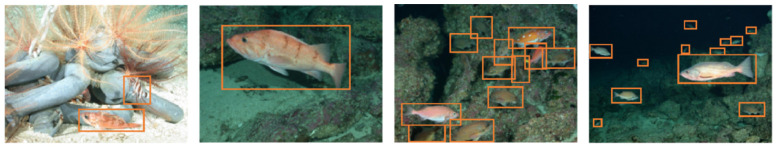
Partial sample of the dataset. The orange box represents the detection box.

The data used in this experiment are collected from a data source and manually labeled, including fish pictures in different backgrounds, covering dark light, noise, small targets and other situations, which is challenging for underwater fish target detection tasks. The raw data come from Labeled Fishes in the Wild [13] provided by the National Oceanic and Atmospheric Administration. This paper filters this dataset and extends annotations for small and ambiguous objects. The experiments in this paper are mainly completed on this dataset. It was converted to the PASCAL VOC dataset format as needed for the investigation. The dataset is described as follows, and some examples are shown in Figure 3.

### 2.2. Single-Stage Underwater Target Detection Algorithm Based on Double Optimization of the Feature Anchor Frame 

The accuracy of traditional one-stage detectors is usually inferior to two-stage detectors. The main reason is that the two-stage detector optimizes the initial anchor boxes through the process of the region recommendation network and generates more refined candidate boxes. However, in single-stage detectors, this process is omitted in pursuit of speed. The single-stage detector presets many a priori boxes on the image at one time to match the target. Therefore, a large number of anchors cause the problem of sample imbalance. To address this issue, RefineDet [14] uses two-stage regression to obtain more accurate results. It filters out a large number of negative anchors through the first classification to balance the positive and negative samples. It then performs anchor box optimization based on the first regression to obtain more accurate results. Although RefineDet performs multiple stages of classification and regression, it is unreasonable to use the same features in two different stages. After the first regression, subsequent operations should focus more on updated anchors and new features. Therefore, AlignDet [15] learns the offset before and after regression through deformable convolution, which solves the problem of feature misalignment to a certain extent. Reappoints [16] uses a weakly supervised method to locate key points and to predict their key-point offsets, which are used as raw feature map offsets for deformable convolutions to align feature maps with object regions.

#### 2.2.1. Composite Connection Backbone Network

Underwater datasets face severe blurring and texture distortion. These problems often affect the quality of features extracted by many relatively shallow backbone networks, thus limiting the discriminative power of classifiers. To this end, there is an urgent need to find backbone networks with more powerful representation capabilities. An important criterion for network design is to enhance the functionality of the basic modules to improve the overall performance of the network. Deeper backbone networks are beneficial for feature extraction, but single-stage detectors focus more on speed advantages. To this end, this chapter first excludes the use of deeper feature extraction backbone networks, as this would slow down single-stage detectors, but redesigning new efficient structures is difficult and labor-intensive. Therefore, according to the existing mainstream feature extraction networks, this chapter explores the relationship between feature extraction of different backbones. Inspired by CBNet [17], this section achieves higher performance than a single backbone by combining existing backbones in the form of compound connections.

**Figure 4:**
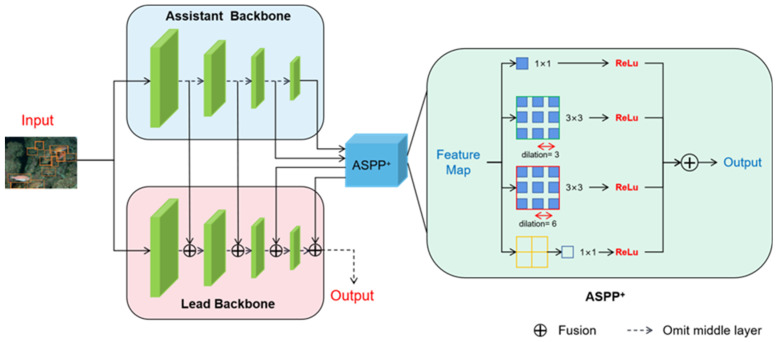
Structure diagram of the backbone network.

The proposed composite connection backbone network is shown in Figure 4, which consists of two parts: composite connection structure and ASPP+.

##### Composite Connection Structure

The left part of Figure 4 shows the composite connection backbone structure. The new backbone network consists of two parts: the main backbone network and the coordinating backbone network. The leading backbone network retains the VGG16 structure in the standard SSD, and the ResNet50 structure is used as a co-backbone network to obtain feature contextual semantic information.

The composite connection backbone network replaces the original network by a combination of basic networks to tap the maximum potential of the existing backbone network. In Figure 4, $F_{l}^{k}$ is defined as the kth main backbone layer, where ∀*k*∊1,....,n−1. $F_{a}^{k}$ represents the kth co-backbone layer. The results of each stage in the co-backbone network can be viewed as higher-level semantic features. In the composite connection backbone network, the output of each stage in the co-backbone network is part of the main backbone network and flows into the next stage after being combined. This way, the fusion of high-level semantic information and low-level visual information can be achieved to generate richer feature representations. The specific process is expressed as the following Formulas (1) and (2):

(1) $$F_{\mathrm{out}\;}^{k}=F_{l}^{k}⊕F_{a}^{k}\;$$

(2) $$F_{\mathrm{OUT}\;}^{k}=\varepsilon \left(F_{\mathrm{out}\;}^{k}\right)$$

Among them, ⊕ represents the process of feature fusion, which defuses features according to the channel axis. $F_{l}^{k}$ and $F_{a}^{k}$ denote the features of the kth stage of the main backbone and the co-backbone, respectively. $F_{out}^{k}$ represents the input value of the subsequent backbone. In the process from $F_{out}^{k}$ to $F_{OUT}^{k}$, ε is defined as the channel compression process of the 1 × 1 convolutional layer.

According to the connection method shown in the Figure 4, the feature maps of the main backbone and the auxiliary backbone, in which layers of the same sequence participate in the fusion, have the same size. Specifically, the 150 × 150, 75 × 75 and 38 × 38 feature maps selected by the backbone network correspond to the output stage feature sizes of ResNet-50. In theory, the connection can be designed to be more complex. It is even possible to choose to fuse feature layers in different orders on the main backbone and co-backbone, and then interpolate linearly to the same size for composite connections.

##### ASPP+

**Figure 5:**
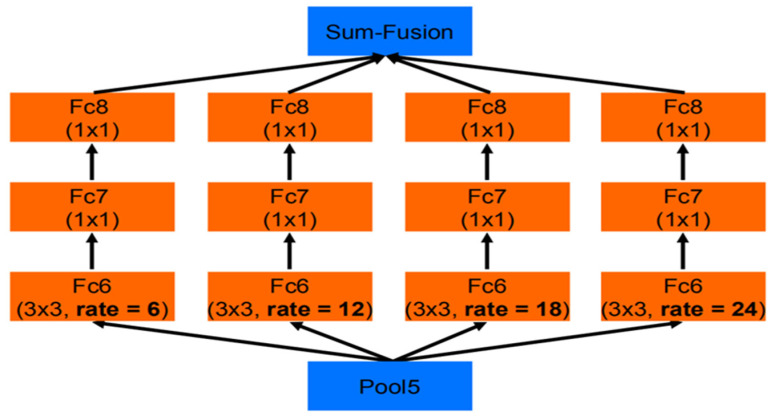
Schematic diagram of ASPP.

The Atrous Spatial Pyramid Pooling module consists of convolution kernels of different sizes represented by a multi-path feature. The right half of Figure 4 shows the enhanced Atrous Spatial Pyramid Pooling (ASPP+) module of this chapter. This module is eventually inserted into the last two layers of the backbone connection. Unlike the original Atrous Spatial Pyramid Pooling, the ASPP+ module takes $F_{a}^{k}$ as input and processes it through four parallel branches. In the first three branches, the multi-scale feature fusion is achieved by combining the atrous convolutional layer and the ReLU layer. The three branches use 1 × 1 convolution, 3 × 3 convolution with a hole rate of 3, and a hole rate of 6. Considering the operation of feature fusion, in order to control the amount of computation, this chapter reasonably allocates the number of channels for each branch. The output channel size of these three branches is set to 1/4 of the number of input channels, and the fourth branch uses a global average pooling layer to compress the features and uses a 1 × 1 kernel to resize the channels to 1/4 of the input. Finally, the features of the four branches will be merged to obtain the output result. Details about the insertion location of this module will be elaborated in subsequent ablation trials. The schematic diagram of ASPP is shown in Figure 5.

#### 2.2.2. Receptive Field Enhancement Module

**Figure 6:**
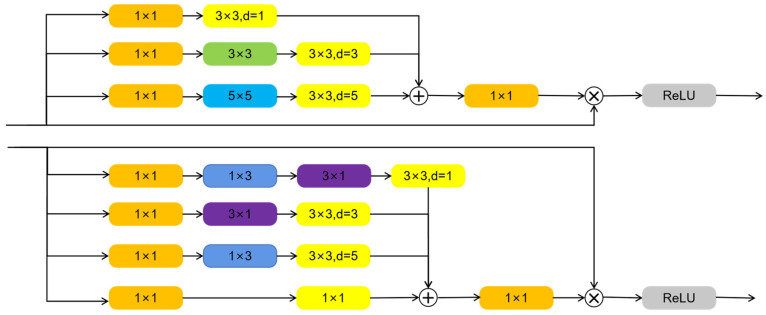
Receptive field enhancement module.

Figure 6 shows the receptive field enhancement module introduced in this section. The upper and lower parts of Figure 6 are the RFA module and the RFA+ module, respectively. The design of the receptive field enhancement module imitates the design idea of ResNet [18] and Inception structure [19], and RFA adopts a multi-path representation structure.

First, multiple branches of the structure process input data in parallel. Each branch consists of a 1 × 1 convolution and several other simple convolutions with kernels of different sizes [20]. Finally, each branch forms a bottleneck-like structure. The kernel size of each branch varies slightly, which is beneficial for capturing multi-scale contextual information. To enlarge the receptive field, atrous convolutions with different dilation rates are used in the final convolutional functional layer to improve the multi-scale detection performance. In this way, more information can be captured on a larger scale while maintaining the same number of parameters [21]. After that, RFA fuses the features of multiple branches and uses 1 × 1 convolution to adjust the channel size. Finally, the receptive field enhancement module also simulates the residual structure using a shortcut connection method, weights the input and summarizes the features of multiple branches to obtain the result [22]. To accommodate various situations, this section proposes two similar structures RFA and RFA+. RFA+ has more branches than RFA and uses many small convolution kernels, which reduces the number of parameters, and is mainly used for shallow layers to enhance small object detection capabilities. RFA+ replaces the 5 × 5 convolution with two stacked 3 × 3 convolutions. This operation not only reduces the computational complexity but also increases the nonlinear capabilities of the model. Furthermore, RFA replaces the original 3 × 3 convolution with 3 × 1 convolution and 1 × 3 convolution. Taking RFA as an example, the whole process can be described by Equation (3):

(3) $$X_{\mathrm{out}\;}=\tau \left(X_{\mathrm{in}\;}⊗\varepsilon \left(Br_{1}⊕Br_{2}⊕Br_{3}\right)\times \;\mathrm{scale}\;\right)$$

Here, $X_{in}$ represents input features. $Br_{k}$ represents different branches, *k*∊1,2,3. ⊕ represent feature fusion. ε represents the process of channel adjustment. The parameter scale represents the linear weight value in the shortcut connection, and the default setting is 0.1. ⨂ represents the feature matrix for element-wise addition. τ represents the final activation function ReLU [23,24].

#### 2.2.3. Predictive Optimization Strategies

The prediction refinement scheme mainly includes two steps: the preprocessing stage and the optimization stage. This process uses a two-step process to refine the prediction of object location and size, which is beneficial for detecting challenging underwater scenes, especially for target groups with large scale variations. The main steps of the prediction optimization scheme are to perform the initial binary classification (front-background classification) and regression in the preprocessing stage and then perform the second classification and regression in the optimization stage to obtain the final prediction result. Unlike RefineDet, the prediction optimization scheme uses six feature prediction layers for refinement (there are only four in RefineDet). In addition, the prediction optimization strategy can make the model more focused on the target through the channel attention mechanism and correct the anchors by learning the offset.

##### Preprocessing Stage

In the preprocessing stage, the prediction results obtained by the receptive field augmentation module (RFA) and additional layers are first processed. Starting from the last layer Conv4_3 of the composite connection backbone, downsampling through additional layers of standard SSD and RFA to achieve the required feature map size for the prediction layer occurs. Conv4_3 is followed by an RFA+ module as the first prediction branch to improve the detection ability of shallow small objects. This paper believes that adding RFA+ to the high-resolution feature map can fully extract the semantic information of the high-resolution feature map; thus, the operation of the high-resolution feature map is beneficial to the detection of small underwater targets. Finally, binary classification and anchor box regression are performed on the information of the six enhanced feature layers [25]. The obvious background boxes are first filtered to provide more high-quality anchor boxes for the refinement stage. The output $C_{1x}$ is used to distinguish between foreground and background. A vector of four-element values $R_{1x}$ is a vector of four-element values used to locate the anchor point [26].

##### Optimization Phase

In the optimization stage, the preprocessing result $C_{1X}$ is first processed by performing a max-pooling operation along the channel axis, and then, it goes through a sigmoid activation function to obtain more salient features. The result of this process is recorded as $S_{1x}$. $S_{1x}$ obtained by max pooling, and the sigmoid function can highlight the location of objects, which is used to enhance the result $X_{out}$ of the six prediction layers. $S_{1x}$ and Xout are multiplied element by element and then added element by element with the value of $X_{out}$ and recorded as $X_{end}$. The prediction optimization module proposed in this section is significantly different from RefineDet, mainly in the double optimization. In theory, the features used in the preprocessing stage should not be the same as the features in the optimization stage [27]. Following this idea, this section also optimizes the front and rear features. Specifically, the TCB connection of RefineDet is replaced by the channel attention mechanism to make the network more focused on the object itself. This process can be represented by the following Formula (4):

(4) $$X_{\mathrm{end}\;}=\left(X_{\mathrm{out}\;}\cdot S_{1x}\right)⊗X_{\mathrm{out}\;}$$

Among them, **·** represents element-by-element multiplication, ⊗ represents element-by-element addition, and $X_{end}$ is the result of the enhancement of the existing position information. The first regression result $R_{1x}$ contains four output vector values: (∆*x*, ∆*y*, ∆*h*, ∆*w*). (∆*x*, ∆*y*) represents the spatial offset of the anchor box center point. (∆*w*, ∆*h*) represents the offset of the anchor box width and height. After that, the additional convolution layer uses (∆*x*, ∆*y*) to calculate the offset of the convolution kernel to correct the sampling center point, correct the anchor frame, and align the anchor frame and the feature. In addition, the optimization stage further enhances the contextual semantic relevance by introducing atrous convolutions in the offset layer. Regarding the classification and regression in the optimization stage, $C_{2x}$ no longer simply performs binary classification, but multi-classification tasks. The output of the deformable convolutional layer is the final result $R_{2x}$. In general, the prediction optimization scheme is similar to RefineDet, but the essence is different. The optimization strategy described in this section not only corrects the anchor box, but also the features.

#### 2.2.4. Loss Function Design and Training

With the proposal of the prediction optimization strategy, the design of the loss function is also fundamentally different from the original SSD loss function. The detection model is optimized by the loss function defined by Equations (5)–(9):

(5) $$L=\frac{1}{N_{pos}^{Pre}}\left(L_{cls}^{Pre}+L_{reg}^{Pre}\right)+\frac{1}{N_{pos}^{Ref}}\left(L_{cls}^{Ref}+L_{reg}^{Ref}\right)$$

(6) $$L_{cls}^{Pre}=\sum_{i}^{n}L_{cls}^{Pre}\left(P_{i},P_{i}^{*}\right)$$

(7) $$L_{reg}^{Pre\;}=\sum_{i\in pos}^{N}L_{reg}^{Pre}\left(t_{i\in pos},p_{i\in pos}^{*}\right)$$

(8) $$L_{cls}^{Ref}=\sum_{i\in pos}^{N}L_{cls}^{Ref}\left(c_{i\in pos},P_{i}^{*}\right)$$

(9) $$L_{reg\;}^{Ref}=\sum_{i\in pos}^{N}L_{reg\;}^{Ref}\left(b_{i\in pos},p_{i\in pos}^{*}\right)$$

In the above formula, *L* is the loss of the final network, $L_{cls}^{Pre}$ and $\;L_{reg}^{Pre}$ represent the classification and regression losses in the preprocessing stage, and $\;L_{cls}^{Ref}$ and $\;L_{reg}^{Ref}$ represent the classification and regression losses in the optimization stage. The classification loss uses the cross-entropy loss function while the regression loss uses the Smooth-L1 loss. $N_{pos}$ represents the number of positive anchor boxes in each link. *i* is the index of anchor boxes in a mini-batch. $P_{i}^{*}$ and $p_{i\in pos}^{*}$ represent the coordinates of the ground-truth label and ground-truth anchor box *i*. $P_{i}$ represents the predicted signature of the anchor box. $t_{i\in pos}$ and $b_{i\in pos}$ are the regression results of the preprocessing stage and the optimization stage, respectively.

## 3. Experiments and Results

The model training in this chapter is all based on the PyTorch deep learning framework, version 1.2.0. The hardware platform is NVIDIA RTX2080Ti, and the neural network model training is accelerated by installing Cuda10.0. During the training phase, the model is trained by default for 160 epochs. A warm-up strategy was used on the initial five epochs, and the learning rate was dynamically selected between −34 × 10 and −610 to slowly approach the initial learning rate of 0.002, and the learning rate changed to a minimum at the 150th epoch [28]. The SGD optimizer was chosen when backpropagating gradient updates. In this section, the detection model backbone extraction network uses a composite connection backbone. The ResNet and VGG networks in the composite connection backbone are loaded with ImageNet pre-training weights, and the input image size is fixed at 300 × 300. The training process uses multi-card training, and the batch-size is set to 32. In addition, the method in this section presets anchor boxes in six prediction layers, and each anchor point presets [4,4,6,6,6,6] anchor boxes. The ratio of anchor boxes is 2:2 and 2:3. In the post-processing stage, non-maximum suppression was used, and the threshold was set to 0.5.

**Figure 7:**
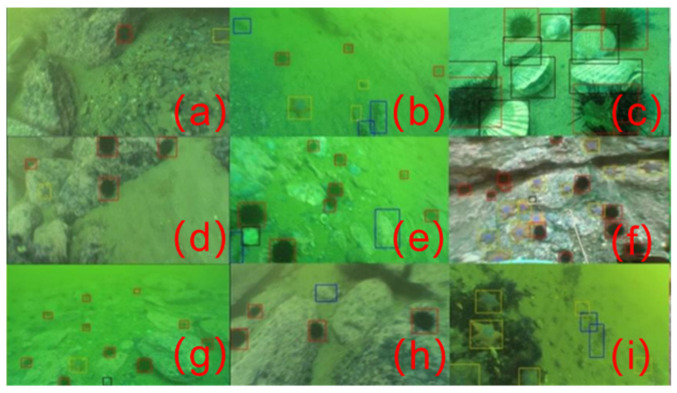
The detection effect of the algorithm in the underwater scene. (**a**–**i**) represent nine different underwater scene detection effects.

Figure 7 shows the detection effect of this algorithm in underwater scenes. The anchor box of each color represents a category, which is marked in the figure. Among them, the black anchor box marks the shell, the red anchor box marks the sea urchin, the blue anchor box marks the sea cucumber, and the yellow anchor box marks the starfish.

First, this section compares the described method (hereafter abbreviated as UWNet) with current mainstream algorithms on the Labeled Fishes in the Wild dataset. Experiments with two input resolutions with input size of 300 × 300 and 512 × 512 were conducted. Table 1 shows the test results of Labeled Fishes in the Wild. It can be seen that UWNet achieves a result of 80.2 mAP on Labeled Fishes in the Wild, which is ahead of most single-stage detectors and even surpasses some two-stage detection algorithms [29]. However, from the data in Table 1, the accuracy of the algorithm still has room for improvement. The main reasons may be as follows: First, the Labeled Fishes in the Wild dataset rarely suffer from blur, occlusion, and scale changes; thus, the algorithm in this paper does not improve much. Second, the detection accuracy will fluctuate according to the version differences of hardware devices and third-party-dependent libraries. UWNet can still improve accuracy while saving some computing resources and time. In conclusion, Table 1 demonstrates that although UWNet does not achieve the current state-of-the-art accuracy, it can still maintain a high detection level in conventional object detection.

Table 2 shows the comparison of the detection accuracy of UWNet on the classic object recognition dataset MS COCO 2015. It can be seen that UWNet achieves the best performance of single-stage detectors. UWNet can achieve an accuracy improvement of 2.1 AP. Careful observation will reveal that the performance of small objects has improved significantly, from 10.9 to 15.1 APs. This benefits from the powerful feature extraction function and the important role of multi-scale functional modules. At an input resolution of 300 × 300, UWNet can provide an accuracy of 32.4 AP.

The predictive optimization schemes are inspired by RefineDet; they are similar but different in nature. The selection and design details of the prediction layer have been explained earlier. This section mainly analyzes the rationality of the number of prediction layers. In RefineDet, four feature layers are selected by the author for prediction, and four prediction layers are selected by experimental analysis to achieve the best accuracy. For underwater datasets, high-level semantic information can help the model to obtain more detailed information about the target, which is helpful for in detecting blurred and distorted images. Therefore, we believe that small feature maps are also necessary for prediction in this experiment. In addition, this experiment performed another small ablation experiment when choosing the number of prediction layers. The accuracies of the four prediction layer structures and the six prediction layer structures are compared [30]. Table 3 shows that each of the six prediction layers outperforms the four prediction layers on the Labeled Fishes in the Wild dataset.

In order to better validate the effectiveness of the proposed method compared with the improved CNN and traditional machine learning methods for four types of underwater target recognition, several typical traditional machine learning methods are designed for underwater target recognition in this paper. The traditional feature extraction methods include Meier frequency cepstrum coefficient (MFCC), Hill (HHT) transform and wavelet transform, etc. The commonly used algorithms include support vector machine (SVM), k-nearest neighbor, BP algorithm and SoftMax regression, etc. A comparison of the experimental results is shown in Table 4.

## 4. Discussion

Due to the existence of a large number of suspended solids in the underwater environment, coupled with the refraction of light and other reasons, underwater imaging generally suffers from color casts, blurring, and uneven illumination. These problems make underwater object detection more challenging. Due to its special application scenarios, underwater target detection not only requires stronger multi-scale and small-target detection capabilities, but also puts forward more stringent requirements for the real-time performance of the model. This paper proposes a single-stage detection method UWNet with double enhancement of anchor boxes and features.

UWNet achieves a result of 80.2 mAP on Labeled Fishes in the Wild dataset, which is ahead of most single-stage detectors and even surpasses some two-stage detection algorithms. However, from the data in Table 1, the accuracy of the algorithm has not yet reached state of the art. The main reasons may be as follows: First, the Labeled Fishes in the Wild dataset rarely suffers from blur, occlusion, and scale changes; thus, the algorithm in this paper does not improve much. Second, the detection accuracy will fluctuate according to the version differences of hardware devices and third-party-dependent libraries. For example, this paper uses RFBNet source code for training, and the detection accuracy can only reach 80.0 mAP. At this time, UWNet is stable at 0.2 mAP higher than RFBNet. Compared with RefineDet, the improvement is 0.2 mAP. However, the input size of this method is 300 × 300, which is smaller than the 320 × 320 input size of RefineDet. UWNet can still improve accuracy while saving some computing resources and time.

UWNet achieves the best performance of single-stage detectors. UWNet can achieve an accuracy improvement of 2.1 AP. Careful observation will reveal that the performance of small objects has improved significantly, from 10.9 to 15.1 APs. This benefits from the powerful feature extraction function and the important role of multi-scale functional modules. At an input resolution of 300 × 300, UWNet can provide an accuracy of 32.4 AP.

Another small ablation experiment was performed while choosing the number of prediction layers. The accuracies of the four prediction layer structures and the six prediction layer structures are compared. Table 3 shows that six prediction layers outperform four prediction layers on both UWD and Labeled Fishes in the Wild datasets.

The double refinement method is obvious for the improvement of the single-stage detection algorithm, but there are some limitations. The improved algorithm based on SSD in this paper is limited by the size of the input image, that is, the current framework in this paper is difficult to use large-resolution images for training. Therefore, its performance is limited, and there are still many deficiencies, which need further in-depth research.

A three-dimensional (3D) surface visualization of the underwater structures, which may be based on a multi-output Gaussian process, can provide a better analysis of the underwater environment. In addition to the UWNet, one may also consider using extreme learning techniques for target detection.

## 5. Conclusions

Aiming at the real-time target detection requirements of underwater scenes, this paper proposes a single-stage detection method with double enhancement of anchor boxes and features. The detection efficiency of this method is high, but the performance is lower than that of the two-stage algorithm due to the redundancy of anchor boxes. Therefore, the feature context relevance is improved by proposing a composite-connected backbone network. The receptive field enhancement module is introduced to improve the multi-scale detection capability. Finally, a prediction refinement strategy is proposed, which refines the anchor frame and features through two regressions, solves the problem of feature anchor frame misalignment, and improves the detection performance of the single-stage underwater algorithm.

## Figures and Tables

**Table 1:** Detection results of Labeled Fishes in the Wild dataset.

Method	Backbone	Input Size	mAP
Two-stage Detectors			
Faster RCNN	VGG16	1000*600	73.2
Faster RCNN	ResNet101	1000*600	76.4
MR-CNN	VGG16	1000*600	78.2
R-FCN	ResNet101	1000*600	80.5
CoupleNet	ResNet101	1000*600	817
One-stage Detectors			
SSD300	VGG16	300*300	77.2
YOLO	GoogleNet	448*448	63.4
YOLOv2	DarkNet19	544*544	78.6
RON^++^	VGG16	320*320	76.6
DSSD	ResNet101	321*321	78.6
RcfineDet	VGG16	320*320	80.0
DES	VGGI6	300*300	79.7
DFPR	VGG16	300*300	79.6
FERNet300	VGG16	300*300	80.2
SSD512	VGG16	512*512	79.5
DSSD512	ResNet101	513*513	81.5
DES512	VGGI6	512*512	81.7
RefineDet512	VGG16	512*512	81.8
DFPR512	VGG16	512*512	81.1
FERNet512	VGGI6	512*512	81.0

**Table 2:** Detection results of MS COCO 2015 dataset.

Method	Backbone	Input Size	Time	AP	AP_50_	AP_75_	AP_S_	AP_m_	AP_l_
Two Stage Detectors									
FasterRCNN	Vgg16	1000*600	147 ms	21.9	42.7	-	-	.	.
CoupleNet	ResNet101	1000*600	121 ms	34.4	54.8	37.2	13.4	38.1	50.8
MaskRCNN	ResNet101	1280*800	210 ms	39.8	62.3	43.4	22.1	43.2	51.2
Single Stage Detectors									
SSD	Vggl6	300*300	20 ms	25.1	43.1	25.8	6.6	25.9	41.4
DSSD	ResNet101	321*321	-	28.0	46.1	29.2	7.4	28.1	47.6
RefineDet	Vggl6	320*320	20 ms	29.4	49.2	31.3	10.0	32.0	44.4
DES	Vggl6	300*300	-	28.3	47.3	29.4	80.5	29.9	45.2
FERNet	Vggl6	300*300	30 ms	32.4	52.6	34.2	15.1	36.3	48.2

**Table 3:** Detection results of different number of prediction layers.

Dataset	Labeled Fishes in the Wild	
Num	4	6
mAP	79.8	80.2

**Table 4:** Recognition rate of underwater targets by different methods.

Identification Method		Recognition Rate (%)
Feature Extraction Method	Classification Method	
MFCC	SVM	83.45
	KNN	76.31
	BP	81.28
	Softmax	85.09
HHT	SVM	83.54
	KNN	82.43
	BP	84.52
	Softmax	83.29
Wavelet transform	SVM	86.95
	KNN	84.36
	BP	86.19
	Softmax	86.75
CNN		89.39
The method proposed in this paper		91.47

## Data Availability

Data confidentiality.
